# Glutathione Transferases as Efficient Ketosteroid Isomerases

**DOI:** 10.3389/fmolb.2021.765970

**Published:** 2021-11-22

**Authors:** Bengt Mannervik, Aram Ismail, Helena Lindström, Birgitta Sjödin, Nancy H. Ing

**Affiliations:** ^1^ Department of Biochemistry and Biophysics, Stockholm University, Stockholm, Sweden; ^2^ Department of Animal Science, Texas A&M AgriLife Research, Texas A&M University, College Station, TX, United States

**Keywords:** ketosteroid isomerase, androstenedione, progesterone, ecdysteroid, glutathione, steroid hormone, enzyme evolution, alternative functions

## Abstract

In addition to their well-established role in detoxication, glutathione transferases (GSTs) have other biological functions. We are focusing on the ketosteroid isomerase activity, which appears to contribute to steroid hormone biosynthesis in mammalian tissues. A highly efficient GST A3-3 is present in some, but not all, mammals. The alpha class enzyme GST A3-3 in humans and the horse shows the highest catalytic efficiency with k_cat_/K_m_ values of approximately 10^7^ M^−1^s^−1^, ranking close to the most active enzymes known. The expression of GST A3-3 in steroidogenic tissues suggests that the enzyme has evolved to support the activity of 3β-hydroxysteroid dehydrogenase, which catalyzes the formation of 5-androsten-3,17-dione and 5-pregnen-3,20-dione that are substrates for the double-bond isomerization catalyzed by GST A3-3. The dehydrogenase also catalyzes the isomerization, but its k_cat_ of approximately 1 s^−1^ is 200-fold lower than the k_cat_ values of human and equine GST A3-3. Inhibition of GST A3-3 in progesterone-producing human cells suppress the formation of the hormone. Glutathione serves as a coenzyme contributing a thiolate as a base in the isomerase mechanism, which also involves the active-site Tyr9 and Arg15. These conserved residues are necessary but not sufficient for the ketosteroid isomerase activity. A proper assortment of H-site residues is crucial to efficient catalysis by forming the cavity binding the hydrophobic substrate. It remains to elucidate why some mammals, such as rats and mice, lack GSTs with the prominent ketosteroid isomerase activity found in certain other species. Remarkably, the fruit fly *Drosophila melanogaster*, expresses a GSTE14 with notable steroid isomerase activity, even though Ser14 has evolved as the active-site residue corresponding to Tyr9 in the mammalian alpha class.

## Introduction

Our understanding of the various physiological roles of the protein family referred to as glutathione transferases (GSTs) has progressed since their discovery in 1961 and continues to evolve. The first publications reported enzyme activity involving displacement of a halide ion from an aromatic compound, resulting in an S-substituted glutathione conjugate (Booth et al., 1961; Combes and Stakelum, 1961). Eric Boyland called the corresponding protein glutathione S-aryltransferase, and further named glutathione S-aralkyltransferase, S-alkyltransferase, S-epoxidetransferase, and S-alkenetransferase on the premise that they were separate enzymes with corresponding substrate specificities (Boyland and Chasseaud, 1969a). However, investigations by several groups (Clark et al., 1973; Habig et al., 1974b; Askelöf et al., 1975) demonstrated that GSTs consists of a family of enzymes with overlapping specificities, such that the original designations became obsolete. These observations led to the simplified name glutathione transferase without any prefix (Mannervik et al., 2005). In spite of their overlap in substrate acceptance, the GSTs demonstrate distinctive activity profiles, as further elaborated below.

GSTs were first recognized as enzymes inactivating xenobiotics, in particular electrophilic mutagens and carcinogens, leading to their excretion as mercapturic acids (Boyland and Chasseaud, 1969b). However, the demonstration that toxic electrophiles produced by endogenous oxidative processes are GST substrates provided a more plausible explanation for driving the evolution of the enzymes (Mannervik, 1985; Mannervik, 1986) than the observation that they catalyze detoxication of foreign compounds.

An independent set of reports identified a remarkably abundant protein in liver with affinity for carcinogens, steroid hormones, bilirubin and synthetic dyes. The wide-ranging binding properties were similar to those of albumin in blood plasma and the name “ligandin” was therefore coined (Litwack et al., 1971). The function of ligandin was proposed to facilitate uptake and transport of hydrophobic molecules such as bilirubin by tight intracellular binding of the ligand (Listowsky et al., 1978). The ligandin protein was identified with GST B in rat liver (Habig et al., 1974a), a dimeric protein composed of subunit A1 in the alpha class, according to the current nomenclature (Mannervik et al., 2005). In the macula of the human eye the pi class member GST P1-1 has more recently been found to be a high-affinity zeaxanthin-binding protein (Bhosale et al., 2004). The physiological role of the carotenoid binding remains unknown, but it was speculated that GST P1-1 may catalyze the double-bond isomerization of lutein into zeaxanthin similar to the ketosteroid isomerization discussed below.

A completely different binding function was uncovered when GST of the pi class was found to be an endogenous inhibitor of Jun N-terminal kinase (JNK), thereby contributing to regulation of a protein kinase responsive to cellular stress (Adler et al., 1999). Members of the alpha and mu classes of GSTs have also been connected to cellular signaling via binding to protein kinases (Board and Menon, 2013).

In addition to the dimeric cytosolic GSTs, which are in focus in the present article, trimeric membrane-associated GST proteins, have also been identified. They are structurally unrelated to the soluble GSTs and the protein family has been named MAPEG (membrane-associated proteins in eicosanoid and glutathione metabolism), since some of the members act as leukotriene C and prostaglandin E synthases (Jakobsson et al., 1999). In this manner MAPEG proteins contribute to the biosynthesis of physiological signal molecules. This is also the function of the soluble sigma class GST S1-1, alternatively known as hematopoietic prostaglandin D synthase (Kanaoka et al., 2000).

### Primary Structures and Classification of Glutathione Transferases

**FIGURE 1:**
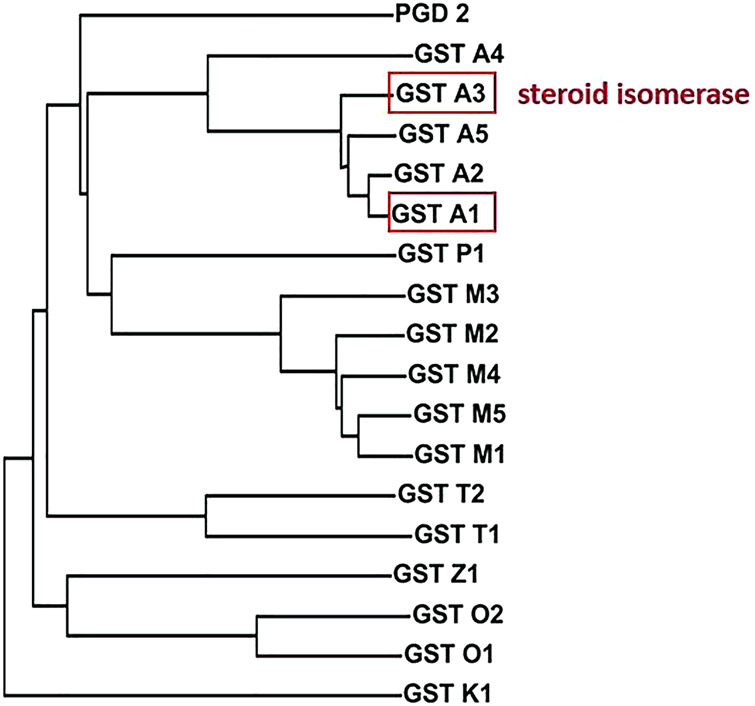
Phylogram of the aminoacid sequences of the 18 subunits of soluble GSTs encoded in the human genome. The members of GST classes alpha, pi, mu, theta, zeta, omega, and kappa are distinguished by corresponding initial letters, whereas the sigma class member GST S1 is commonly referred to as prostaglandin D2 synthase (PGD 2). The catalytically active forms are dimers with broad but distinct specificities for alternative substrates. Prominent ketosteroid isomerase activity with 5-and rosten-3,17-dione and 5-pregnen-3,20-dione is displayed by GST A3-3, and approximately 10-fold lower by GST A1-1, but not by other alpha class members or another GST.

40 years ago scattered aminoacid sequence data were available for rodent and human GSTs. In combination with immunological cross-reactivities and functional data they formed the basis of an evolutionary tree (Mannervik et al., 1985). Three separate branches of mammalian cytosolic GSTs named alpha, mu, and pi were recognized, which clearly were distinct from the microsomal GST. Additional divergent classes of cytosolic GSTs were subsequently identified and named omega, sigma, theta, and zeta as well as the distantly related kappa class GST found in mitochondria and peroxisomes (Hayes et al., 2005). Figure 1 shows a phylogram based on the aminoacid sequences of the 18 distinct gene products occurring as soluble dimeric proteins in humans. The current nomenclature specifies a particular GST by the first letter of the name of the class that the GST belongs to, followed by its subunit composition by number in the class (Mannervik et al., 2005). When the biological species needs to be specified a prefix is added, e.g., HsaGST A1-2 for the *Homo sapiens* GST composed of subunit 1 and subunit 2 in the alpha class. Members of the same GST class show high aminoacid identity in their sequences, whereas members of different classes usually show <40% identity.

In the original analysis, classification appeared to be supported by functional data such as substrate selectivity and inhibition characteristics (Mannervik et al., 1985). However, the finding that mutation of a single aminoacid residue can drastically alter the substrate specificity and other characteristics (Björnestedt et al., 1995b; Norrgård et al., 2006) makes these criteria less conclusive for GST class assignments. Prominent ketosteroid isomerase activity is displayed by HsaGST A3-3, and approximately 10-fold lower by HsaGST A1-1, but not by the other alpha class GSTs (Johansson and Mannervik, 2001). The effect of subtle differences in sequence makes it difficult to accurately predict functional properties, as evidenced by the quest for GSTs with ketosteroid isomerase activity described below.

### Discovery of Ketosteroid Isomerase

In 1955 Talalay and Wang discovered a new enzymatic reaction involving ketosteroids, including 5-androsten-3,17-dione (5-AD) and 5-pregnen-3,20-dione (5-PD), and purified a highly active isomerase from *Pseudomonas* bacteria (Talalay and Wang, 1955).

**FIGURE 2:**
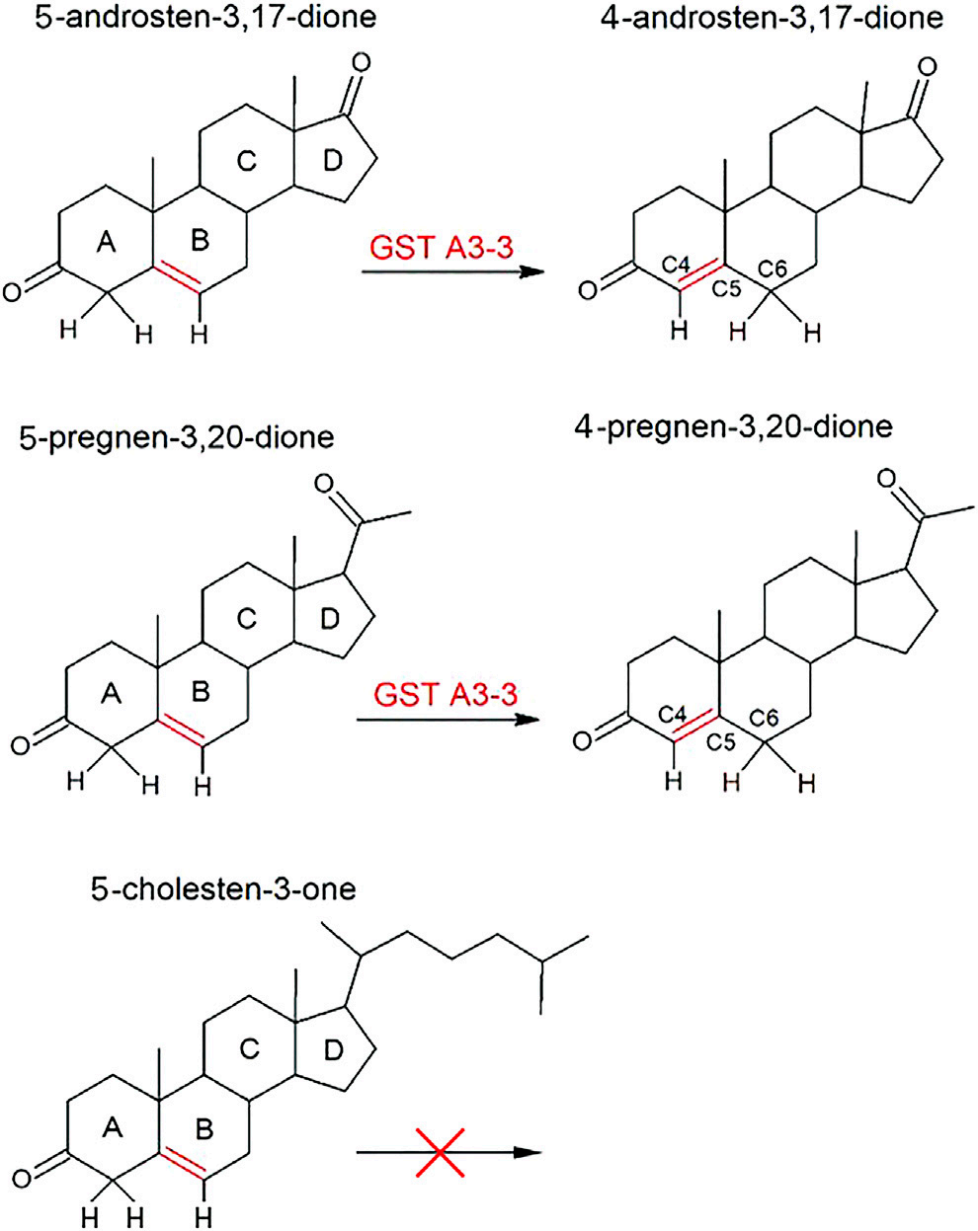
Double-bond isomerization of 5-androsten-3,17-dione and 5-pregnen-3,20-dione catalyzed by GST A3-3 and other ketosteroid isomerases. The structurally related 5-cholesten-3-one is not a substrate.

The enzyme, ketosteroid isomerase, obtained from several bacterial species has subsequently been extensively investigated by incisive structural and functional studies owing to its extraordinary catalytic efficiency (Yabukarski et al., 2020; and papers cited therein). The chemical reaction involves the deprotonation of C4 in the A ring of the steroid substrate, thereby enabling the double bond between C5 and C6 to isomerize to a double bond between C4 and C5 (Figure 2). The process proceeds via an intermediate dienolate and is independent of metal ions or any other cofactor.

In 1976 Benson and Talalay discovered that the cytosolic fraction of rat liver catalyzes the same steroid isomerization of 5-androsten-3,17-dione, but in distinction from the bacterial enzyme the reaction included reduced glutathione as a designated coenzyme (Benson and Talalay, 1976). Another difference was the modest specific activity of the rodent enzyme (0.545 μmol min^−1^ mg^−1^) in comparison with the five orders of magnitude higher activity of the *Pseudomonas* enzyme (Kawahara et al., 1962). Further investigations identified the hepatic rat enzyme with GST B (nowadays called RnoGST A1-1) and it was furthermore found that purified human hepatic GSTs from the Jakoby laboratory displayed even higher specific activity than the rat enzyme (Benson et al., 1977).

In retrospect, it is clear that the ketosteroid isomerase activity of the GST in human liver (Benson et al., 1977) resided in the alpha class subunit A1, which occurs in the two isoenzymes GST A1-1 and GST A1-2 (called GST B_1_B_1_ and B_1_B_2_ in Stockman et al., 1985). The members of the different GST classes are encoded on separate chromosomes and the Board laboratory identified an alpha class gene on chromosome 6, which corresponded to a protein not previously recognized (Suzuki et al., 1993). The new enzyme GST A3-3 was cloned from human placenta and characterized in our laboratory and found to be the most efficient ketosteroid isomerase so far detected in mammals (Johansson and Mannervik, 2001). Notably, the enzyme was not detected in liver, but only in steroid hormone producing tissues. More recently, an equally efficient horse GST A3-3 was identified in steroidogenic organs such as ovary, testis, and adrenal gland (Lindström et al., 2018).

### Mechanism of Ketosteroid Isomerase Catalyzed by Mammalian Glutathione Transferases

The double-bond isomerization of ketosteroids has been investigated with 5-AD and 5-PD (Figure 2). In both substrates the double bond is shifted towards the 3-keto group thereby forming a conjugated π-bond system in the products 4-AD and 4-PD. Remarkably, 5-cholesten-3-one, derived by oxidation of the 3-hydroxyl group of cholesterol, is not a substrate in spite of a structure of the A and B rings identical to that in 5-AD and 5-PD, in which the double bond isomerization occurs (Figure 2). The inability of 5-cholesten-3-one to serve as substrate for the bacterial ketosteroid isomerase was noted in the original publication (Talalay and Wang, 1955), and the purified mammalian GST A3-3 from different sources also lack detectable activity with this steroid (unpublished work in our laboratory). Structural modeling suggests that the bulky aliphatic sidechain prevents binding to the active site.

Investigations of the *Pseudomonas* ketosteroid isomerase based on deuterium and tritium labeling of reactants provided evidence for direct and stereospecific diaxial proton transfer from the 4β to the 6β position (Talalay, 1965). On the other hand, tritium exchange with the medium suggested that the isomerase occurring in animal tissues did not catalyze a similar direct proton transfer. Experiments with purified GSTs have not been reported, but the mechanism involving the sulfur of glutathione (see below) could be expected to show proton exchange with the solvent.

The role of glutathione in the mammalian GSTs catalyzing the ketosteroid isomerization was not evident, since the bacterial isomerase did not require a cofactor (Benson and Talalay, 1976). Glutathione could be either a direct participant in the reaction mechanism or just serve as an activator of the GST. Like most GST-catalyzed chemical reactions the isomerization of 5-AD takes place spontaneously at a low, but measurable rate. Glutathione stimulates this nonenzymatic reaction, but addition of GST enzyme increases the rate by several orders of magnitude (Pettersson and Mannervik, 2001; Johansson and Mannervik, 2002). The GST in the absence of glutathione also shows a minor catalytic effect on the steroid double-bond isomerization. S-Methylglutathione, which is lacking the free thiol group present in glutathione, stimulates neither the nonenzymatic nor the GST-catalyzed isomerization, but inhibits the glutathione-independent GST catalysis (Pettersson and Mannervik, 2001). These findings are rationalized by the direct involvement of glutathione as a coenzyme. Detailed steady-state kinetic analyses of human GST A1-1 clarified the overall mechanism involving the two main functional residues, the thiolate of glutathione and the phenol group of Tyr9. The pK_a_ value of the thiol of glutathione is lowered by 2.5 units from 9.2 in solution to 6.7 when bound to GST A1-1 (Pettersson and Mannervik, 2001). The thiolate serves as base removing a proton from C4 in the steroid substrate. The sulfur atom of glutathione is close to the OH of Tyr9 in the active site (Grahn et al., 2006), but the hydroxyl group of Tyr9 has only a marginal effect on the ionization of glutathione as demonstrated by the small increase of its pK_a_ value from 6.7 to 7.2 when Tyr9 was mutated into Phe (Pettersson and Mannervik, 2001). Instead, the basic group causing the unusual acidity of the thiol group resides in the glutathione molecule itself in the form of the free α-carboxylate of the glutamyl residue (Gustafsson et al., 2001). The absence of the carboxylate in 4-aminobutyrylcysteinylglycine (GABA-Cys-Gly) lowers the ketosteroid isomerase activity by a factor of 24,000. Structural studies demonstrate that the binding mode of the alternative thiol substrate is similar to that of glutathione (Grahn et al., 2006). Even though Tyr9 is not a base causing the unusual acidity of the thiol, it contributes to the mechanism as indicated by the low isomerase activity of the Tyr9Phe mutant, being only 2% of the wildtype activity (Pettersson and Mannervik, 2001). The pK_a_ of Tyr9 is 8.1 in the free GST A1-1, but the value increases to 9.2 when glutathione is bound (Björnestedt et al., 1995a). The increased pK_a_ appears dependent on the ionized thiolate, since S-methylglutathione is without effect on the ionization of Tyr9.

A second aminoacid residue of mechanistic importance is Arg15, which is widely conserved among the known alpha class GSTs (Dourado et al., 2010). Its guanidinium sidechain can form a hydrogen bond to the sulfur of glutathione, thereby lowering the pK_a_ value of the thiol group, and mutation of Arg15 to Ala or His decreases the steroid isomerase activity by two orders of magnitude, twice the effect of the Tyr9Phe mutation (Björnestedt et al., 1995a).

The mechanistic studies of human GST A1-1 were extended to human GST A3-3 following the discovery of the latter enzyme (Johansson and Mannervik, 2001). The results were very similar, even though glutathione bound to GST A3-3 showed a thiol pK_a_ value of 6.1 and Tyr9 phenol pK_a_ of 7.9, somewhat lower than the corresponding pK_a_ values of GST A1-1 (Johansson and Mannervik, 2002). The specific activity with 5-androsten-3,17-dione decreased 350-fold by the Tyr9Phe mutation due to the same diminution of the k_cat_ value. A limited effect on the pK_a_ value of the glutathione thiol by the same mutation suggested that the evolutionarily conserved Arg15 makes a major contribution to ionization of the thiol group.

**FIGURE 3:**
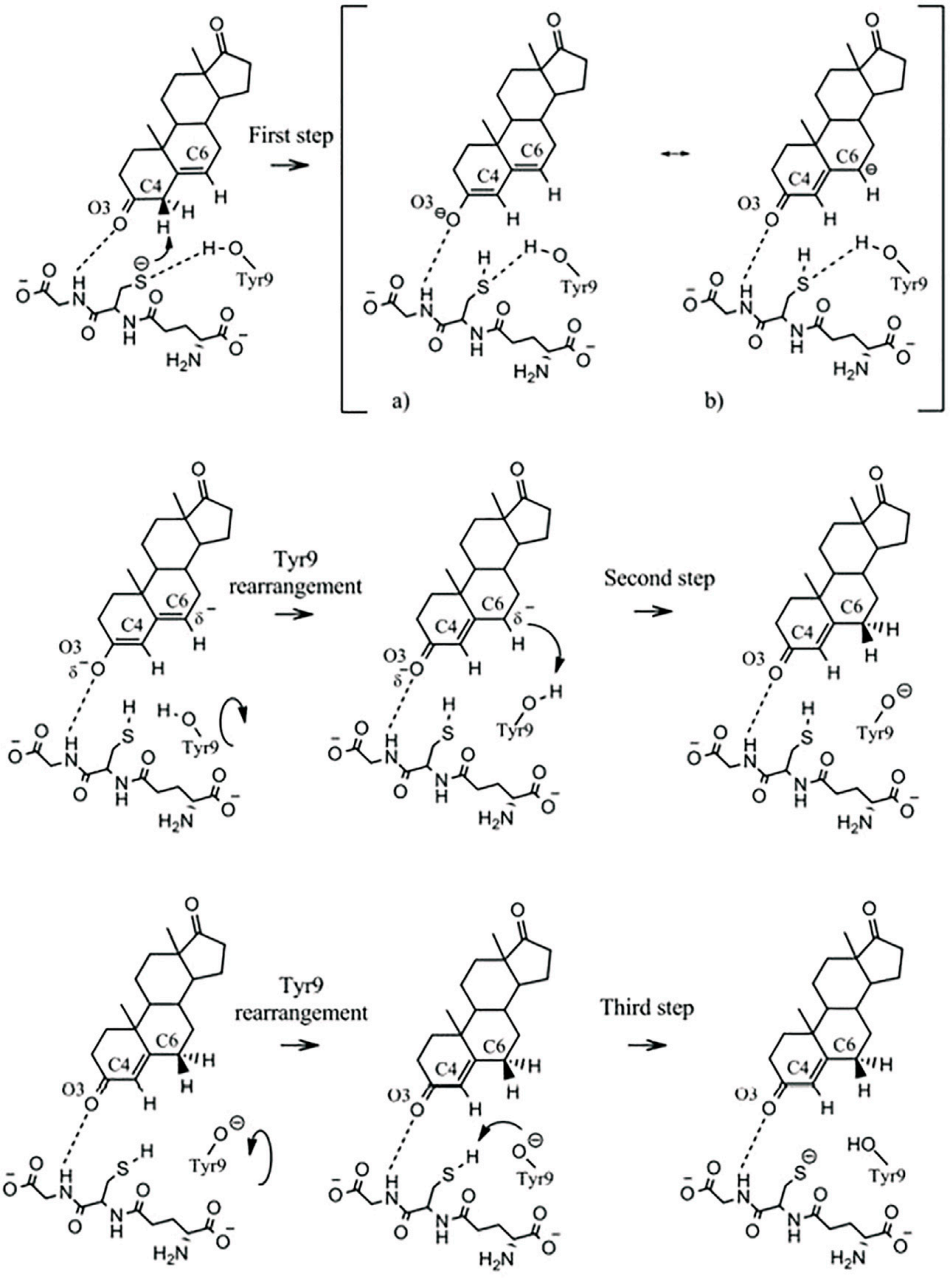
Mechanism proposed for the isomerization of 5-androsten-3,17-dione by GST A3-3. Formation of the thiolate, which acts as a base, requires the α-carboxylate of Glu in the glutathione molecule. The NH of Gly in glutathione polarizes the O=C3 bond of the steroid. The hydroxyl group of Tyr9 in the active site rotates between the sulfur of glutathione and C6 of the steroid substrate and serves as an acid in the catalysis. (Figure with permission from Dourado et al., 2014).

Human GST A3-3, which, similar to the equine GST A3-3, shows the highest ketosteroid isomerase activity in mammals, has been subjected to analysis at the atomic level based on density functional theory calculations to put forward a refined mechanism for the isomerization of 5-AD (Dourado et al., 2014). The mechanism includes three consecutive chemical steps interspaced by two conformational rearrangements of Tyr9, comprising a total of five elementary steps (Figure 3).

In the first step, the thiolate of glutathione acts as a base and deprotonates carbon C4. The second step is a conformational rearrangement of Tyr9 leading to a direct interaction of Tyr9 with carbon C6. In the third step, Tyr9 serves as an acid and protonates C6 in the second chemical step. The fourth step of the mechanism consists of a second Tyr9 rearrangement that allows direct interaction of the Tyr9 phenolate with the glutathione thiol. In the fifth step of the mechanism, which is the third chemical step, Tyr9 deprotonates the glutathione thiol, restoring the active site of the enzyme to the initial state. In the computational study, no support was found for the hypothesis that a water molecule could serve as an alternative to Tyr9 as a proton donor (Dourado et al., 2014). Glutathione has a twofold function in the catalysis. In addition to serving as a base, it polarizes the O3 atom of the steroid substrate by a hydrogen bond from the nitrogen of glycine in the glutathione molecule. This bond promotes the formation of the dienolate intermediate in the isomerization. The alternative cofactor γ-Glu-Cys, which is lacking glycine, shows 6.5-fold lower catalytic efficiency than glutathione in the isomerization of 5-AD catalyzed by EcaGST A3-3 (Lindström et al., 2018). This decrease corresponds to a 1.1 kcal mol^−1^ loss of binding energy due to the absence of the glycine residue. For comparison of the mechanisms, the reaction catalyzed by the bacterial ketosteroid isomerase entails stabilization of the dienolate by a Tyr and an Asp residue interacting with the O_3_ atom, whereas a single Asp residue serves the acid/base function exerted by the glutathione thiolate/Tyr9 pair in GST A3-3 (Pollack, 2004).

### Substrate Selectivity in Glutathione Transferases

**FIGURE 4:**
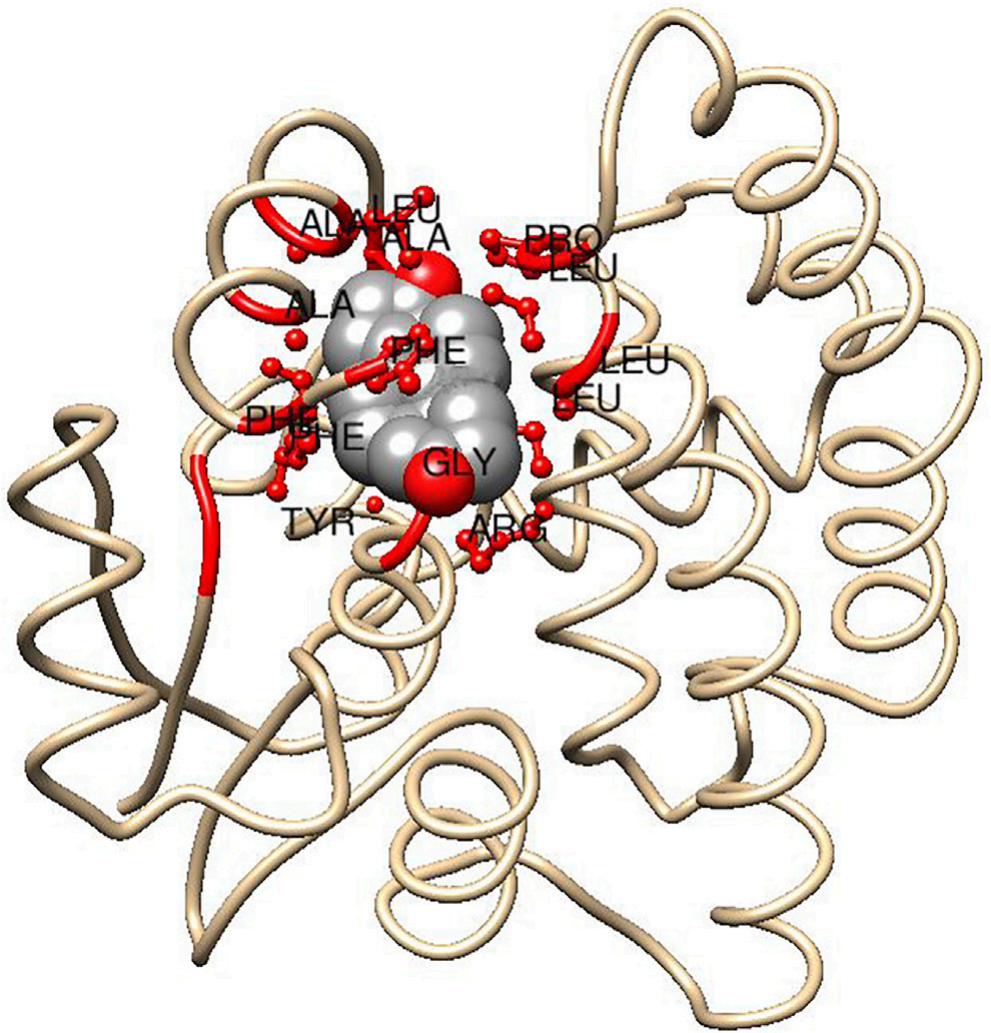
Subunit of human GST A3-3 indicating H-site interactions with a bound steroid ligand. Fragments <5.0 Å distant from the steroid molecule are indicated in red, including ball and stick representations of side chains from (counter clockwise): Phe10, Tyr9, Gly14, and Arg15 (below); Leu107, Leu108, Pro110, and Leu111 (right); Ala208, Leu213, Ala216, Phe220, and Phe222 (left). The model is based on the crystal structure of GST A3-3 (PDB ID: 2vcv) in complex with the product 4-androsten-3,17-dione using Chimera 1.15 (Pettersen et al., 2004). It is assumed that the substrate 5-androsten-3,17-dione binds in a similar pose subject to the conformational adjustments for the interchange of the sp^3^ and sp^2^ hybridizations of C4 and C6 in the two isomeric steroid molecules (cf. Figure 2).

The cytosolic mammalian GSTs are composed of two similar or identical subunits, which in the alpha class usually each contain 222 encoded residues. The N-terminal domain consisting of 80-odd residues adopts a thioredoxin-like fold and harbors the G-site, which binds glutathione with a number of highly conserved residues as well as with ionic bonds from Asp101 and Glu 131 in the adjacent subunit (Sinning et al., 1993). A second binding cavity, the H-site, is formed by three regions of the primary structure: 1) in the N-terminus contributions from residues 10,12 and the methylene groups of Arg13, Gly14, and Arg15; 2) in the middle of the sequence residues 107, 108, 110, 111 and the aliphatic portion of Glu104 (which forms an ionic bond with Arg15); and 3) in the C-terminus by residues 208, 213, 216, 220, and 222 (Figure 4). The H-site is highly hydrophobic and variations in its topology are important for the substrate selectivity of the enzyme. Modest structural H-site rearrangements in HsaGST A1-1 can install a novel pronounced preference for alkenal substrates (Nilsson et al., 2000). Similarly, HsaGST A2-2, which has minimal ketosteroid isomerase activity was mutated in five H-site residues to attain a 3,500-fold higher catalytic efficiency with 5-AD (Pettersson et al., 2002).

### Development of Ketosteroid Isomerase Activity in the Glutathione Transferase Structure

The discovery of human GST A3-3 (HsaGST A3-3) and its association with steroidogenic tissues (Johansson and Mannervik, 2001) prompted the search for mammalian orthologs with similar outstanding catalytic efficiency. The equine EcaGST A3-3 and HsaGST A3-3 display k_cat_/K_m_ values of 1.6 × 10^7^ and 8.6 × 10^6^ M^−1^s^−1^, respectively (Lindström et al., 2018) ranking them among the enzymes with particularly high activity (Wolfenden and Snider, 2001), even though the bacterial ketosteroid isomerase has an even higher catalytic efficiency of approximately 4 × 10^8^ M^−1^s^−1^ (Hawkinson et al., 1991) approaching a diffusion-controlled encounter rate of enzyme and substrate (10^8^ -10^9^ M^−1^s^−1^).

BtaGSTA1-1, an alpha class GST identified in tight association with bovine steroidogenically active cells and shown to be hormonally regulated by gonadotropins in the bovine ovarian follicles, appeared to be a likely isomerase (Rabahi et al., 1999). However, in spite of 82% identity in primary structure with human GST A3-3, the bovine enzyme showed two orders of magnitude lower activity with both 5-AD and 5-PD (Raffalli-Mathieu et al., 2007). By contrast, a porcine enzyme entitled SscGST A2-2, linked to ovarian follicular differentiation and showing 84% sequence identity with the human enzyme, was found to approach the high ketosteroid activity of HsaGST A3-3 (Fedulova et al., 2010). Likewise, CjaGST A3-3 from the common marmoset was shown to have high activity with the steroid substrates (Ismail et al., 2021). In an attempt to find additional GSTs with prominent ketosteroid isomerase activity, mRNAs from the testes of dog, goat, and gray short-tailed opossum were probed for homologs of the horse and human GSTA3 sequences (Hubert et al., 2020). The resultant novel GSTA3 mRNA and deduced protein sequences had a high level of conservation with the human GSTA3 mRNA and protein sequences (≥70% and ≥64% identities, respectively). However, in spite of conservation of the majority of the H-site aminoacid residues expected to interact with the steroid, none of the expressed proteins were found to display high isomerase activity. No GST characterized in the rat or mouse has been found to display more than 2% of the high ketosteroid isomerase activity found in HsaGST A3-3 (Mannervik and Danielson, 1988; Hayes and Pulford, 1995).

Based on mutational studies of human GSTs the five H-site residues in positions 10, 12, 111, 208, and 216 were judged to be of particular importance for the ketosteroid isomerase activity (Johansson and Mannervik, 2002; Pettersson et al., 2002). However, comparison of the H-site residues of GSTs displaying high activity with those having low activity does not identify a signature set of residues conferring the high rates of ketosteroid isomerization (Table 1).

**TABLE 1:** Aminoacid residues in the H-site of some mammalian GSTs. Species designations: Bta, *Bos taurus* (cow); Cja, *Callithrix jacchus* (common marmoset); Clu, *Canis lupus* (dog); Eca, *Equus caballus* (horse); Hsa, *Homo sapiens* (man); Ssc, *Sus scrofa* (pig). Five positions judged to govern ketosteroid isomerase activity based on mutational studies (Johansson and Mannervik, 2002; Pettersson et al., 2002) are underlined.

Position	EcaGST A3-3	EcaGST A1-1	HsaGST A3-3	HsaGST A1-1	HsaGST A2-2	SscGST A2-2	SscGST A1-1	CjaGST A3-3	CjaGST A1-1	BtaGST A1-1
10	F	F	F	F	S	F	F	F	F	F
12	G	A	G	A	I	G	G	A	A	G
14	G	G	G	G	G	G	G	G	G	G
104	E	E	E	E	E	E	E	E	E	E
107	L	M	L	L	L	L	L	L	L	M
108	L	Y	L	L	L	L	L	L	L	H
110	P	P	P	P	P	P	P	P	P	P
111	I	M	L	V	F	L	L	L	F	L
208	G	L	A	M	M	T	M	T	T	T
213	V	I	L	L	L	L	L	V	L	I
216	S	A	A	A	S	A	A	A	A	A
220	F	F	F	F	F	F	F	F	F	F
222	F	F	F	F	F	F	I	F	F	F

The catalytic efficiency of HsaGST A3-3 with 5-AD is 5000-fold higher than that of HsaGST A2-2, and we found that mutating the five residues in the H-site of HsaGST A2-2 into the corresponding HsaGST A3-3 residues enhanced the efficiency to 70% of the HsaGST A3-3 value (Pettersson et al., 2002). Structural analysis of the wild-type HsaGST A2-2 and HsaGST A3-3 shows that the steroid substrate is not binding to HsaGST A2-2 in an orientation compatible with ketosteroid isomerase activity, apparently explaining the different activities of the two enzymes (Tars et al., 2010). The primary structures of the two enzymes differ in 20 additional positions outside the H-site, but these differences appear to have minimal influence on the measured activities. In spite of the insights gained from mutational analyses, it has proved difficult to predict which of the largely conserved H-sites in different GSTs that would support high ketosteroid isomerase activity (Table 1). The dog CluGST A3-3 protein, differing in only four of the residues forming the substrate-binding site in HsaGST A3-3 and EcaGST A3-3, displays a specific activity with 5-AD of only 1.1 μmol min^−1^ mg^−1^, 200-fold lower than the specific activities of the human and equine enzymes (A. Ismail, unpublished).

A static representation of the topology and electrostatic field in the H-site (Warshel, 1998) will have to be complemented with a description of the dynamics of the entire molecule (Hammes et al., 2011), and possibly invoke effects of the adjacent subunit of the dimeric enzyme for a deeper insight into structural requirements for the isomerization mechanism.

### Substrate Discrimination in Promiscuous Enzymes

**FIGURE 5:**
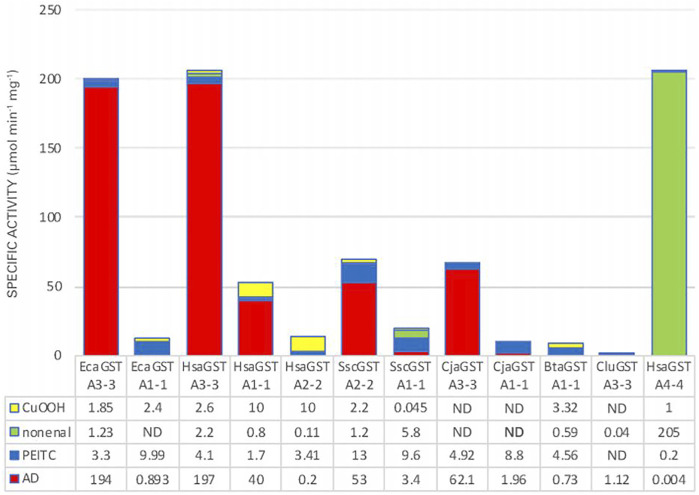
Stacked specific activities with four physiologically relevant substrate categories. CuOOH, cumene hydroperoxide; nonenal, *trans*-2-nonenal; PEITC, phenethyl isothiocyanate; AD, 5-androsten-3,17-dione. The specific activities are given in units of μmol min^−1^ mg^−1^ and compiled from EcaGST A3-3 (Lindström et al., 2018); EcaGST A1-1 (A. Ismail, unpublished); HsaGST A3-3 and HsaGST A1-1 (Johansson and Mannervik, 2001); HsaGST A2-2 (Johansson and Mannervik, 2001; Zhang et al., 2012); SscGST A2-2 (Fedulova et al., 2010); SscGST A1-1 (Fedulova et al., 2011); CjaGST A3-3 and CjaGST A1-1 (Ismail et al., 2021); BtaGST A1-1 (Raffalli-Mathieu et al., 2007); CluGST A3-3 (A. Ismail, unpublished); HsaGST A4-4 (Hubatsch et al., 1998).

GSTs, like many other enzymes involved in detoxication, are promiscuous such that they can act on several different substrates and catalyze many alternative chemical reactions. This broad substrate acceptance enables one particular enzyme to catalyze the biotransformation of numerous toxicants. However, if a catalyst evolves for a more specific function, it may be advantageous to suppress the activity with alternative substrates. Even though GSTs can act on hundreds of electrophilic compounds (Chasseaud, 1979), the majority of them have no physiological significance and therefore lack importance in the evolution of substrate selectivity. The development of steroid isomerase activity should therefore be evaluated in relation to reactions with other substrates that may occur in tissues. We have therefore scored specific activities of alpha class GSTs with cumene hydroperoxide (CuOOH) as an organic hydroperoxide representing the peroxidase activity of GSTs (Seeley et al., 2006), *trans*-2-nonenal representing reactive α,β-unsaturated aldehydes formed by lipid peroxidation (Berhane et al., 1994), and phenethyl isothiocyanate (PEITC) representing dietary exposure to isothiocyanates in cruciferous vegetables (Zhang et al., 1995). Figure 5 shows stacked specific activities with these substrates combined with the specific activity with 5-AD. Clearly, the human HsaGST A3-3 and the equine EcaGST A3-3 stand out as the enzymes with the most prominent ketosteroid isomerase activities, but also HsaGST A1-1, the porcine SscGST A2-2, and the marmoset CjaGST A3-3 have high intrinsic activities with 5-AD. It should be noted that the absolute values should be considered in relation to the cellular concentration of the GST, such that the approximately 3- to 4-fold lower specific activities of SscGST A2-2, CjaGST A3-3, and HsaGST A1-1, if required, could be compensated for by a higher expression level of the protein. Therefore, HsaGST A1-1, which is abundantly present in liver, kidney, and small intestine would make an important contribution to the capacity of steroid isomerization in these tissues.

**FIGURE 6:**
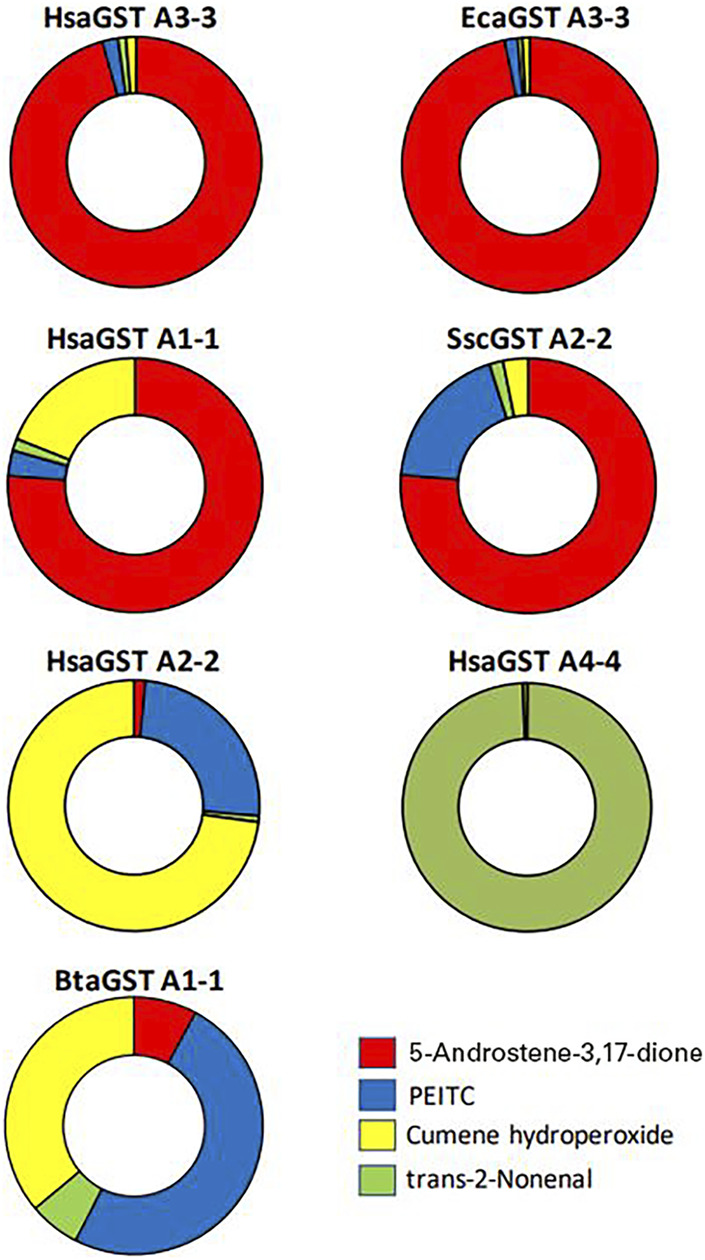
Substrate selectivity profiles of some mammalian GSTs with four physiologically relevant substrates including the steroid 5-androsten-3,17-dione. The doughnut profiles show the fractions of the total sum of catalytic activities with four alternative substrates representing physiologically relevant substrates. HsaGST A3-3 and EcaGST A3-3 demonstrate strong bias in favor of the isomerization of the steroid substrate, and HsaGST A1-1 and SscGST A2-2 also have an obvious preference for 5-AD. HsaGST A2-2 and BtaGST A1-1 are dominated by the activities with the hydroperoxide and the isothiocyanate PEITC, whereas HsaGST A4-4 is highly specific for the alkenal.

From the evolutionary perspective the relative activities with the alternative substrates of importance should be considered. Figure 6 illustrates the differences in selectivities among some of the GSTs with the four selected substrates. The donut diagrams are based on the activities when the enzymes operate in the standard assay systems. The discrimination among the substrates almost exclusively favor 5-AD for HsaGST A3-3 and EcaGST A3-3, whereas *trans*-2-nonenal is a signature substrate for HsaGST A4-4. These illustrations are suggestive of evolutionary selection for activities with these substrates. For a more stringent quantitative analysis of substrate discrimination, the ambient substrate concentrations should also be considered. It is well known that an enzyme simultaneously presented with two alternative substrates will catalyze their reactions in the ratio between their respective catalytic efficiency multiplied with the ambient substrate concentration, k_cat_/K_m__×_ [substrate]. However, substrate concentrations will vary over time, such that relevant values would be difficult to assess.

### Physiological Role of Ketosteroid Isomerase

**FIGURE 7:**
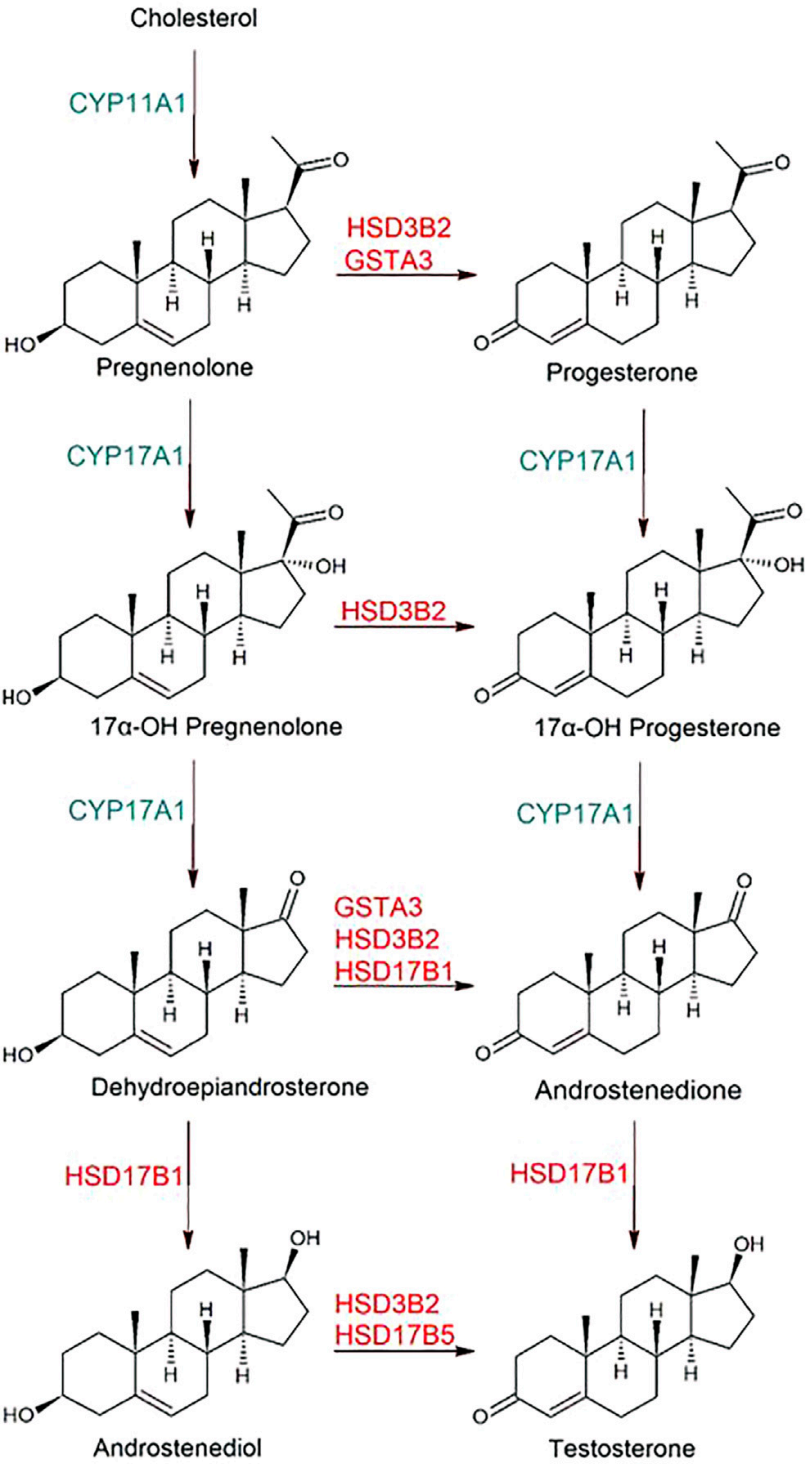
Biosynthetic reactions leading from cholesterol to the steroid hormones progesterone and testosterone. The chemical transformations in the left column represent the delta5-pathway and those in the right column the delta4-pathway. Genes indicated are encoding enzymes involved in the processes those so far identified by transcriptome analysis during the pregnancy of mares (Loux et al., 2020). CYP, cytochrome P450; GST, glutathione transferase; HSD, hydroxysteroid dehydrogenase. The 3-keto intermediates derived by oxidation of the 3-hydroxy groups of 17α-hydroxypregnenolone and androstenediol may also be GST substrates, but they have not yet been tested. Further reactions from progesterone to corticosteroids, and from androstedione and testosterone to estrogens are not depicted.

Steroid hormone production is key to regulation of numerous physiological functions. Originating from the cholesterol molecule, various biochemical transformations lead divergently to a large number of end products, which exert their actions via nuclear receptors and in some cases via receptors on cell membranes. The series of biochemical transformations begins with the sidechain cleavage catalyzed by the cytochrome P450 enzyme CYP11A1 to give pregnenolone (Figure 7). Further reactions catalyzed by CYP17A1 lead to 17α-hydroxypregnenolone and dehydroepiandrosterone. Finally, 17β-hydroxysteroid dehydrogenase HSD17B1 can reduce dehydroepiandrosterone to androstenediol. In all these compounds the C3 hydroxyl group and the C5-C6 double bond originating in cholesterol are conserved. This metabolic route is referred to as the delta5-pathway. However, each of the four products can be oxidized to 3-ketosteroids with a concomitant double bond isomerization to give progesterone, 17α-hydroxyprogesterone, androstenedione, and testosterone, respectively (Figure 7), and the alternative route to the sex hormones is named the delta4-pathway. The 3-hydroxy oxidations are catalyzed by NAD^+^-dependent hydroxysteroid dehydrogenases of which 3β-hydroxysteroid dehydrogenase 2 (HSD3B2) is the most prevalent in gonads and adrenal gland. A second isoenzyme (HSD3B1) is abundant in placenta, mammary gland and prostate. It is also reported that the 17β-hydroxysteroid dehydrogenases HSD17B1, HSD17B5, and HSD17B7 catalyze the C3 hydroxy oxidation (Hilborn et al., 2017).

The double-bond isomerization following 3-hydroxy oxidation in the steroids is conventionally ascribed to an ancillary function of the dehydrogenase, displaying 3-ketosteroid isomerase activity in addition to the redox catalysis. The two human 3β-hydroxysteroid dehydrogenases have been investigated in detail by Thomas and coworkers (e.g., Thomas et al., 2005), and their studies indicate that the 3-ketosteroid 5-AD formed by oxidation of dehydroepiandrosterone with NAD^+^ as a coenzyme remains as an intermediate bound to the enzyme prior to its isomerization to 4-AD. The NADH generated in the preceding oxidation appears to be required for the activation of the isomerase activity of the same enzyme molecule. The k_cat_ values for the isomerase reaction catalyzed by the human HSD3B1 and HSD3B2 enzyme have been reported as 50.2 and 81.5 min^−1^ (i.e., 0.84 and 1.36 s^−1^). By contrast, the k_cat_ values of the ketosteroid isomerase activity of human, equine, and marmoset GST A3-3 are two orders of magnitude higher, ranging between 204 and 261 s^−1^ (Ismail et al., 2021).

**FIGURE 8:**
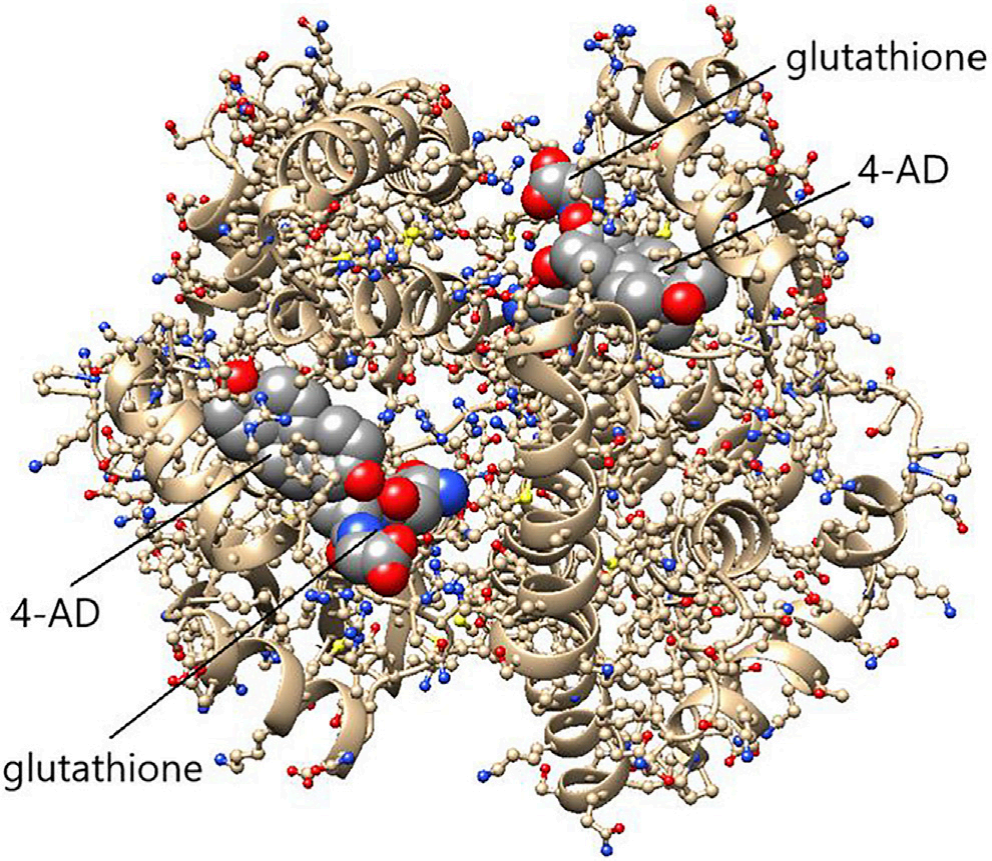
Structure of the efficient ketosteroid isomerase HsaGST A3-3 in a ternary complex with 4-androsten-3,17-dione (4-AD) and glutathione. The dimeric protein shows 4-AD bound in the H-site of each subunit in close proximity to glutathione in the G-site. The substrate 5-AD is assumed to bind essentially in the same pose as the product 4-AD shown. The structure indicates that the bulky substituent on C17 prevents 5-cholesten-3-one from binding as a substrate. (Figure based on the crystal structure PDB ID: 2vcv).

Our enzymological studies have established 5-PD and 5-AD as outstanding substrates for the equine and human GST A3-3 enzymes, and we predict that the corresponding 3-keto intermediates derived by oxidation of the 3-hydroxy groups of 17α-hydroxypregnenolone and androstenediol will also be substrates, although they have not yet been tested. As noted above, 5-cholesten-3-one is not showing any activity with the mammalian enzymes, presumably due to steric hindrance by the bulky sidechain (Figure 8).

A recent investigation of the equine transcriptome in chorioallantois and endometrium at several time points throughout gestation has established GST A3-3 among the actors in endocrinology (Loux et al., 2020). Figure 7 shows enzymes identified in the horse via their transcripts. In tissues, the ketosteroid isomerase reactions catalyzed by GSTs are strictly dependent on dehydrogenases such as 3β-hydroxysteroid dehydrogenase and 17β-hydroxysteroid dehydrogenase for production of the 3-keto substrates from the corresponding 3-hydroxysteroid precursors, since the GSTs are lacking dehydrogenase activity. The GSTs appear to supplement the isomerase activity of the dual function dehydrogenases. The importance of GST A3-3 is shown by inhibiting the enzyme with drugs or by RNA interference of GST A3-3 biosynthesis, treatments which both suppress the hormone production in two steroidogenic human cell lines (Raffalli-Mathieu et al., 2008). It was also noted that the promoter region of the GSTA3 gene contains three putative binding sites for steroidogenic factor 1 (SF-1), which serves as a master regulator of endocrine functions. SF-1 transduction in human mesenchymal stem cells induces cell differentiation into steroidogenic cell lineages. Using this differentiation system SF-1 was indeed shown to regulate the expression of human alpha class genes in parallel with HSD3B2 (Matsumura et al., 2013). These results provide evidence that HsaGSTA3-3, and also HsaGST A1-1, catalyze steroid isomerization in coordination with HSD3B2 to produce progesterone and 4-androstenedione from their delta5-precursors pregnenolone and dehydroepiandrosterone in steroidogenic cells.

Further evidence for the physiological relevance of the GST isomerase activity derives from the coregulation of dehydrogenase and GSTA3 gene transcription in the horse (Loux et al., 2020). In general, animals differ in their patterns of steroids, receptors, and synthetic routes, but it is noteworthy that the features of human and equine endocrinology show many similarities in pregnancy, parturition, and premature delivery (Conley, 2016). The discovery that HsaGST A3-3 and EcaGST A3-3 both show higher ketosteroid isomerase activity than orthologs in other species investigated is an additional similarity.

### Future Prospects

The available data suggest that GSTs have a supportive function, which can prevent the overproduction of the 3-keto delta5-steroids formed by hydroxysteroid dehydrogenases and thereby prevent product inhibition. In spite of data from cell studies, the quantitative importance of the GST contribution in living organisms is not known. Most likely, the influence will change with the activity of the steroidogenic processes. Conclusive studies in intact organisms have not been reported, partly in anticipation of a suitable animal model. Rodents such as rats and mice do not express a GST with high ketosteroid isomerase activity. Horse and pig have highly active GSTs, but are large animals not well suited for relevant experiments. Humans would not be investigated until animal data have been obtained. Possibly, the common marmoset could be considered as a model (Ismail et al., 2021).

Elucidation of the effects at the physiological level may lay the groundwork for medical applications to numerous conditions that are dependent on steroid hormones (Hubert et al., 2020). CRISPR/Cas9 (Ran et al., 2013) and other refined techniques of molecular genetics could be applied to clarify the roles of discrete proteins in the network of steroidogenic reactions.

**FIGURE 9:**
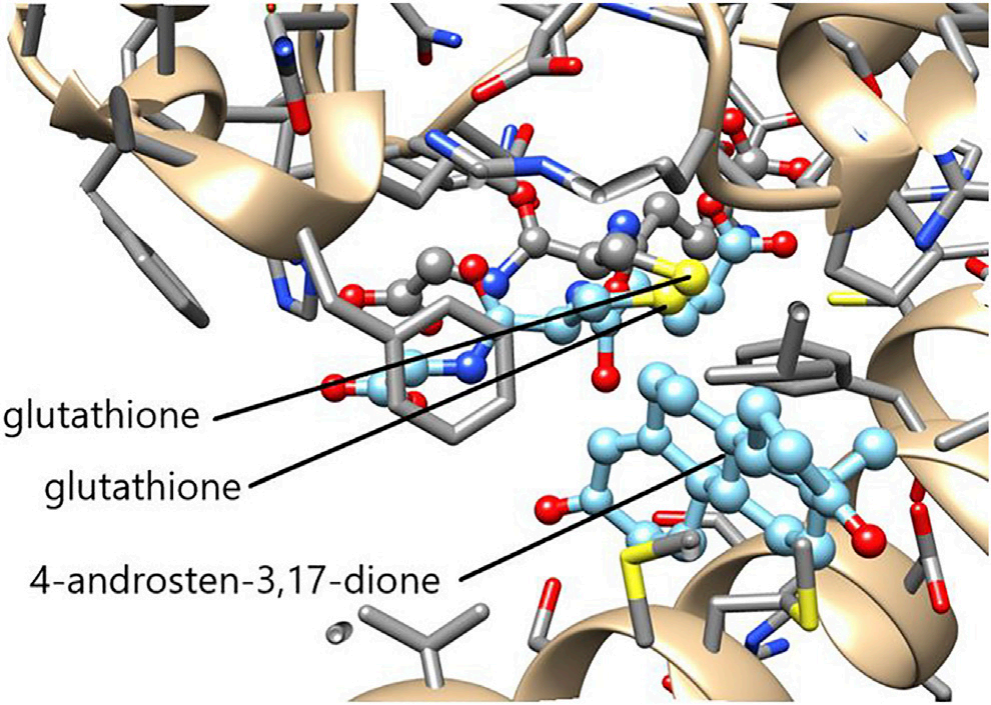
Superimposed active sites of GSTE14 from D. melanogaster and HsaGST A3-3. The crystal structures of GSTE14 (PBD ID: 6t2t) and HsaGST A3-3 (PDB ID: 2vcv) were matched in Chimera (Pettersen et al., 2004). The aminoacid residues of GSTE14 are rendered in gray sticks and the bound glutathione colored by element. The ligands glutathione and 4-androsten-3,17-dione in light blue are the only structures shown from HsaGST A3-3. Note the similar locations of the reactive sulfur (yellow) of glutathione near the scissile bonds of the steroid in the two enzymes.

The function of GSTs in steroid metabolism in not limited to mammalian organisms, but has also been discovered in insects. The epsilon class GSTE14 in the fruit fly *Drosophila melanogaster* plays an essential, but as yet mechanistically undefined, role in ecdysteroid hormone biosynthesis (Chanut-Delalande et al., 2014; Enya et al., 2014). Ecdysteroids are formed from dietary cholesterol and are essential to molting (ecdysis) and formation of the exoskeleton. The accepted biosynthetic pathway is initiated by 7,8-dehydrogenation of cholesterol and is terminated by a series of reaction catalyzed by different cytochrome P450 enzymes leading to ecdysone and 20-hydroxyecdysone. In 2014 two research groups identified the gene product of *Noppera-bo* as GSTE14 in *D*. *melanogaster* (Chanut-Delalande et al., 2014; Enya et al., 2014). Like deficient cytochrome P450 genes, null alleles of *Noppera-bo* result in embryonic lethality, embryonic cuticle abnormalities, and decreased ecdysteroid concentrations. Numerous other Dipteran and Lepidopteran insects have been shown to encode an apparently orthologous GST of the epsilon class (Koiwai et al., 2020). The epsilon class of GSTs is not present in mammals and the active-site Tyr9 crucial to the mammalian ketosteroid isomerase activity is replaced by a Ser residue, but the insect GST still displays considerable activity in the double-bond isomerization of 5-AD and 5-PD (Škerlová et al., 2020). A superposition of the crystal structures of GSTE14 and HsaGST A3-3 shows that the sulfur of glutathione is positioned adjacent to the reactive atoms in the androstenedione molecule in a potentially functional pose (Figure 9).

The endogenous substrate of the insect GST is unidentified, and neither 5-AD and 5-PD are known to occur in insects. The conservation of the orthologs to GSTE14 in other insects attests to an essential function, which merits further biochemical investigations.

### Concluding Remarks

The soluble GSTs demonstrate admirable versatility as catalysts of chemical reactions encompassing aromatic and aliphatic substitution, Michael addition, transacylation, transnitrosylation, carbamoylation, reduction, and isomerization, and it has been demonstrated that modest structural modifications can drastically transmute the substrate selectivity profile (Blikstad et al., 2008). In fact, successive single mutations can significantly alter the profile such that an evolutionary trajectory can navigate the ambient multidimensional substrate environment (Zhang et al., 2012). It has been argued that Darwinian enzyme evolution operates via an ensemble called quasi-species rather than selecting an individual “best” mutant (Mannervik et al., 2009). Manifestation of the quasi-species is dependent on the substrate matrix, implying that a dormant activity, such as steroid isomerization, will only become overt if the relevant substrate is present and the reaction provides selective advantage (Kurtovic et al., 2008). In general, it has become increasingly apparent that many proteins are multi-tasking and display “moonlighting” functions (Jeffery, 2020). The ketosteroid isomerase activity of GSTs exemplify how detoxication enzymes have acquired novel functions in the biochemistry of steroids and contributed to the expanding functional network of glutathione transferases.

## References

[B1] Adler V. Yin Z. Fuchs S. Y. Benezra M. Rosario L. Tew K. D. (1999). Regulation of JNK Signaling by GSTp. EMBO J. 18 (5), 1321–1334. 10.1093/emboj/18.5.1321 10064598 PMC1171222

[B2] Askelöf P. Guthenberg C. Jakobson I. Mannervik B. (1975). Purification and Characterization of Two Glutathione S-Aryltransferase Activities from Rat Liver. Biochem. J. 147 (3), 513–522. 10.1042/bj1470513 810139 PMC1165479

[B3] Benson A. M. Talalay P. Keen J. H. Jakoby W. B. (1977). Relationship between the Soluble Glutathione-dependent delta 5-3-ketosteroid Isomerase and the Glutathione S-Transferases of the Liver. Proc. Natl. Acad. Sci. 74 (1), 158–162. 10.1073/pnas.74.1.158 264670 PMC393217

[B4] Benson A. M. Talalay P. (1976). Role of Reduced Glutathione in the Δ5-3-ketosteroid Isomerase Reaction of Liver. Biochem. biophysical Res. Commun. 69 (4), 1073–1079. 10.1016/0006-291x(76)90482-4 6023

[B5] Berhane K. Widersten M. Engström A. Kozarich J. W. Mannervik B. (1994). Detoxication of Base Propenals and Other Alpha, Beta-Unsaturated Aldehyde Products of Radical Reactions and Lipid Peroxidation by Human Glutathione Transferases. Proc. Natl. Acad. Sci. 91 (4), 1480–1484. 10.1073/pnas.91.4.1480 8108434 PMC43183

[B6] Bhosale P. Larson A. J. Frederick J. M. Southwick K. Thulin C. D. Bernstein P. S. (2004). Identification and Characterization of a Pi Isoform of Glutathione S-Transferase (GSTP1) as a Zeaxanthin-Binding Protein in the Macula of the Human Eye. J. Biol. Chem. 279 (47), 49447–49454. 10.1074/jbc.M405334200 15355982

[B7] Björnestedt R. Stenberg G. Widersten M. Board P. G. Sinning I. Alwyn Jones T. (1995a). Functional Significance of Arginine 15 in the Active Site of Human Class Alpha Glutathione Transferase A1-1. J. Mol. Biol. 247 (4), 765–773. 10.1016/s0022-2836(05)80154-8 7723030

[B8] Björnestedt R. Tardioli S. Mannervik B. (1995b). The High Activity of Rat Glutathione Transferase 8−8 with Alkene Substrates Is Dependent on a Glycine Residue in the Active Site. J. Biol. Chem. 270 (50), 29705–29709. 10.1074/jbc.270.50.29705 8530359

[B9] Blikstad C. Shokeer A. Kurtovic S. Mannervik B. (2008). Emergence of a Novel Highly Specific and Catalytically Efficient Enzyme from a Naturally Promiscuous Glutathione Transferase. Biochim. Biophys. Acta (Bba) - Gen. Subjects 1780 (12), 1458–1463. 10.1016/j.bbagen.2008.07.007 18706975

[B10] Board P. G. Menon D. (2013). Glutathione Transferases, Regulators of Cellular Metabolism and Physiology. Biochim. Biophys. Acta (Bba) - Gen. Subjects 1830 (5), 3267–3288. 10.1016/j.bbagen.2012.11.019 23201197

[B11] Booth J. Boyland E. Sims P. (1961). An Enzyme from Rat Liver Catalysing Conjugations with Glutathione. Biochem. J. 79 (3), 516–524. 10.1042/bj0790516 16748905 PMC1205680

[B12] Boyland E. Chasseaud L. F. (1969a). Glutathione S-Aralkyltransferase. Biochem. J. 115 (5), 985–991. 10.1042/bj1150985 5360727 PMC1185241

[B13] Boyland E. Chasseaud L. F. (1969b). The Role of Glutathione and Glutathione S-Transferases in Mercapturic Acid Biosynthesis. Adv. Enzymol. Relat. areas Mol. Biol. 32, 173–219. 10.1002/9780470122778.ch5 4892500

[B14] Chanut-Delalande H. Hashimoto Y. Pelissier-Monier A. Spokony R. Dib A. Kondo T. (2014). Pri Peptides Are Mediators of Ecdysone for the Temporal Control of Development. Nat. Cel Biol 16 (11), 1035–1044. 10.1038/ncb3052 25344753

[B15] Chasseaud L. F. (1979). The Role of Glutathione and Glutathione S-Transferases in the Metabolism of Chemical Carcinogens and Other Electrophilic Agents. Adv. Cancer Res. 29, 175–274. 10.1016/s0065-230x(08)60848-9 474272

[B16] Clark A. G. Smith J. N. Speir T. W. (1973). Cross-specificity in Some Vertebrate and Insect Glutathione-Transferases with Methyl Parathion (Dimethyl P-Nitrophenyl Phosphorothionate), 1-Chloro-2,4-Dinitrobenzene and S-Crotonyl-N-Acetylcysteamine as Substrates. Biochem. J. 135 (3), 385–392. 10.1042/bj1350385 4772267 PMC1165840

[B17] Combes B. Stakelum G. S. (1961). A Liver Enzyme that Conjugates Sulfobromophthalein Sodium with Glutathione*. J. Clin. Invest. 40, 981–988. 10.1172/jci104337 13694895 PMC290815

[B18] Conley A. J. (2016). Review of the Reproductive Endocrinology of the Pregnant and Parturient Mare. Theriogenology 86 (1), 355–365. 10.1016/j.theriogenology.2016.04.049 27156685

[B19] Dourado D. F. A. R. Fernandes P. A. Mannervik B. Ramos M. J. (2010). Glutathione Transferase A1-1: Catalytic Importance of Arginine 15. J. Phys. Chem. BB 114 (4), 1690–1697. 10.1021/jp908251z 20052987

[B20] Dourado D. F. A. R. Fernandes P. A. Mannervik B. Ramos M. J. (2014). Isomerization of Δ5-Androstene-3,17-dione into Δ4-Androstene-3,17-dione Catalyzed by Human Glutathione Transferase A3-3: A Computational Study Identifies a Dual Role for Glutathione. J. Phys. Chem. AA 118 (31), 5790–5800. 10.1021/jp410810q 24739064

[B21] Enya S. Ameku T. Igarashi F. Iga M. Kataoka H. Shinoda T. (2014). A Halloween Gene Noppera-Bo Encodes a Glutathione S-Transferase Essential for Ecdysteroid Biosynthesis via Regulating the Behaviour of Cholesterol in Drosophila. Sci. Rep. 4, 6586PMC4192634. 10.1038/srep06586 25300303 PMC4192634

[B22] Fedulova N. Raffalli-Mathieu F. Mannervik B. (2011). Characterization of Porcine Alpha-Class Glutathione Transferase A1-1. Arch. Biochem. Biophys. 507 (2), 205–211. 10.1016/j.abb.2010.12.015 21172301

[B23] Fedulova N. Raffalli-Mathieu F. Mannervik B. (2010). Porcine Glutathione Transferase Alpha 2-2 Is a Human GST A3-3 Analogue that Catalyses Steroid Double-Bond Isomerization. Biochem. J. 431 (1), 159–167. 10.1042/BJ20100839 20673231

[B24] Grahn E. Novotny M. Jakobsson E. Gustafsson A. Grehn L. Olin B. (2006). New crystal Structures of Human Glutathione Transferase A1-1 Shed Light on Glutathione Binding and the Conformation of the C-Terminal helix. Acta Crystallogr. D Biol. Cryst. 62 (Pt 2), 197–207. 10.1107/S0907444905039296 16421451

[B25] Gustafsson A. Pettersson P. L. Grehn L. Jemth P. Mannervik B. (2001). Role of the Glutamyl α-Carboxylate of the Substrate Glutathione in the Catalytic Mechanism of Human Glutathione Transferase A1-1. Biochemistry 40 (51), 15835–15845. 10.1021/bi010429i 11747461

[B26] Habig W. H. Pabst M. J. Fleischner G. Gatmaitan Z. Arias I. M. Jakoby W. B. (1974a). The Identity of Glutathione S-Transferase B with Ligandin, a Major Binding Protein of Liver. Proc. Natl. Acad. Sci. 71 (10), 3879–3882. 10.1073/pnas.71.10.3879 4139704 PMC434288

[B27] Habig W. H. Pabst M. J. Jakoby W. B. (1974b). Glutathione S-Transferases. J. Biol. Chem. 249 (22), 7130–7139. 10.1016/s0021-9258(19)42083-8 4436300

[B28] Hammes G. G. Benkovic S. J. Hammes-Schiffer S. (2011). Flexibility, Diversity, and Cooperativity: Pillars of Enzyme Catalysis. Biochemistry 50 (48), 10422–10430. 10.1021/bi201486f 22029278 PMC3226911

[B29] Hawkinson D. C. Eames T. C. M. Pollack R. M. (1991). Energetics of 3-oxo-.DELTA.5-steroid Isomerase: Source of the Catalytic Power of the Enzyme. Biochemistry 30 (45), 10849–10858. 10.1021/bi00109a007 1932007

[B30] Hayes J. D. Flanagan J. U. Jowsey I. R. (2005). Glutathione Transferases. Annu. Rev. Pharmacol. Toxicol. 45, 51–88. 10.1146/annurev.pharmtox.45.120403.095857 15822171

[B31] Hayes J. D. Pulford D. J. (1995). The Glut Athione S-Transferase Supergene Family: Regulation of GST and the Contribution of the Lsoenzymes to Cancer Chemoprotection and Drug Resistance Part I. Crit. Rev. Biochem. Mol. Biol. 30 (6), 445–520. 10.3109/10409239509083491 8770536

[B32] Hilborn E. Stål O. Jansson A. (2017). Estrogen and Androgen-Converting Enzymes 17β-Hydroxysteroid Dehydrogenase and Their Involvement in Cancer: with a Special Focus on 17β-Hydroxysteroid Dehydrogenase Type 1, 2, and Breast Cancer. Oncotarget 8 (18), 30552–30562. 10.18632/oncotarget.15547 28430630 PMC5444764

[B33] Hubatsch I. Ridderström M. Mannervik B. (1998). Human Glutathione Transferase A4-4: an Alpha Class Enzyme with High Catalytic Efficiency in the Conjugation of 4-hydroxynonenal and Other Genotoxic Products of Lipid Peroxidation. Biochem. J. 330 (Pt 1), 175–179. 10.1042/bj3300175 9461507 PMC1219124

[B34] Hubert S. M. Samollow P. B. Lindström H. Mannervik B. Ing N. H. (2020). Conservation of Glutathione S-Transferase mRNA and Protein Sequences Similar to Human and Horse Alpha Class GST A3-3 across Dog, Goat, and Opossum Species. bioRxiv. 10.1101/2020.11.25.396168 PMC10526480 37759820

[B35] Ismail A. Sawmi J. Mannervik B. (2021). Marmoset Glutathione Transferases with Ketosteroid Isomerase Activity. Biochem. Biophys. Rep. 27, 101078. 10.1016/j.bbrep.2021.101078 34286113 PMC8280513

[B36] Jakobsson P.-J. Morgenstern R. Mancini J. Ford-Hutchinson A. Persson B. (1999). Common Structural Features of Mapeg-A Widespread Superfamily of Membrane Associated Proteins with Highly Divergent Functions in Eicosanoid and Glutathione Metabolism. Protein Sci. 8 (3), 689–692. 10.1110/ps.8.3.689 10091672 PMC2144274

[B37] Jeffery C. J. (2020). Enzymes, Pseudoenzymes, and Moonlighting Proteins: Diversity of Function in Protein Superfamilies. Febs J. 287 (19), 4141–4149. 10.1111/febs.15446 32534477

[B38] Johansson A.-S. Mannervik B. (2002). Active-site Residues Governing High Steroid Isomerase Activity in Human Glutathione Transferase A3-3. J. Biol. Chem. 277 (19), 16648–16654. 10.1074/jbc.M201062200 11872752

[B39] Johansson A.-S. Mannervik B. (2001). Human Glutathione Transferase A3-3, a Highly Efficient Catalyst of Double-Bond Isomerization in the Biosynthetic Pathway of Steroid Hormones. J. Biol. Chem. 276 (35), 33061–33065. 10.1074/jbc.M104539200 11418619

[B40] Kanaoka Y. Fujimori K. Kikuno R. Sakaguchi Y. Urade Y. Hayaishi O. (2000). Structure and Chromosomal Localization of Human and Mouse Genes for Hematopoietic Prostaglandin D Synthase. Eur. J. Biochem. 267 (11), 3315–3322. 10.1046/j.1432-1327.2000.01362.x 10824118

[B41] Kawahara F. S. Wang S.-F. Talalay P. (1962). The Preparation and Properties of Crystalline Δ5-3-Ketosteroid Isomerase. J. Biol. Chem. 237, 1500–1506. 10.1016/s0021-9258(19)83730-4 14454546

[B42] Koiwai K. Inaba K. Morohashi K. Enya S. Arai R. Kojima H. (2020). An Integrated Approach to Unravel a Crucial Structural Property Required for the Function of the Insect Steroidogenic Halloween Protein Noppera-Bo. J. Biol. Chem. 295 (20), 7154–7167. 10.1074/jbc.RA119.011463 32241910 PMC7242711

[B43] Kurtovic S. Shokeer A. Mannervik B. (2008). Emergence of Novel Enzyme Quasi-Species Depends on the Substrate Matrix. J. Mol. Biol. 382 (1), 136–153. 10.1016/j.jmb.2008.07.003 18640124

[B44] Lindström H. Peer S. M. Ing N. H. Mannervik B. (2018). Characterization of Equine GST A3-3 as a Steroid Isomerase. J. Steroid Biochem. Mol. Biol. 178, 117–126. 10.1016/j.jsbmb.2017.11.011 29180167

[B45] Listowsky I. Gatmaitan Z. Arias I. M. (1978). Ligandin Retains and Albumin Loses Bilirubin Binding Capacity in Liver Cytosol. Proc. Natl. Acad. Sci. 75 (3), 1213–1216. 10.1073/pnas.75.3.1213 274712 PMC411440

[B46] Litwack G. Ketterer B. Arias I. M. (1971). Ligandin: a Hepatic Protein Which Binds Steroids, Bilirubin, Carcinogens and a Number of Exogenous Organic Anions. Nature 234 (5330), 466–467. 10.1038/234466a0 4944188

[B47] Loux S. C. Conley A. J. Scoggin K. E. El-Sheikh Ali H. Dini P. Ball B. A. (2020). New Insights in Equine Steroidogenesis: an In-Depth Look at Steroid Signaling in the Placenta. Reproduction (Cambridge, England) 160 (1), 65–82. 10.1530/REP-20-0015 32408268

[B48] Mannervik B. Ålin P. Guthenberg C. Jensson H. Tahir M. K. Warholm M. (1985). Identification of Three Classes of Cytosolic Glutathione Transferase Common to Several Mammalian Species: Correlation between Structural Data and Enzymatic Properties. Proc. Natl. Acad. Sci. United States America 82 (21), 7202–7206. 10.1073/pnas.82.21.7202 PMC390817 3864155

[B49] Mannervik B. Board P. G. Hayes J. D. Listowsky I. Pearson W. R. (2005). Nomenclature for Mammalian Soluble Glutathione Transferases. Methods Enzymol. 401, 1–8. 10.1016/S0076-6879(05)01001-3 16399376

[B50] Mannervik B. Danielson U. H. (1988). Glutathione Transferases-Sstructure and Catalytic Activity. CRC Crit. Rev. Biochem. 23 (3), 283–337. 10.3109/10409238809088226 3069329

[B51] Mannervik B. (1986). Glutathione and the Evolution of Enzymes for Detoxication of Products of Oxygen Metabolism. Chem. Scripta 26B, 281–284.

[B52] Mannervik B. Runarsdottir A. Kurtovic S. (2009). Multi-substrate-activity Space and Quasi-Species in Enzyme Evolution: Ohno's Dilemma, Promiscuity and Functional Orthogonality. Biochem. Soc. Trans. 37 (Pt 4), 740–744. 10.1042/BST0370740 19614586

[B53] Mannervik B. (1985). The Isoenzymes of Glutathione Transferase. Adv. Enzymol. Rel. Areas Mol. Biol. 57, 357–417. 10.1002/9780470123034.ch5 3898742

[B54] Matsumura T. Imamichi Y. Mizutani T. Ju Y. Yazawa T. Kawabe S. (2013). Human Glutathione S-Transferase A (GSTA) Family Genes Are Regulated by Steroidogenic Factor 1 (SF-1) and Are Involved in Steroidogenesis. FASEB J. : official Publ. Fed. Am. Societies Exp. Biol. 27 (8), 3198–3208. 10.1096/fj.12-222745 23650189

[B55] Nilsson L. O. Gustafsson A. Mannervik B. (2000). Redesign of Substrate-Selectivity Determining Modules of Glutathione Transferase A1-1 Installs High Catalytic Efficiency with Toxic Alkenal Products of Lipid Peroxidation. Proc. Natl. Acad. Sci. United States America 97 (17), 9408–9412. 10.1073/pnas.150084897 PMC16877 10900265

[B56] Norrgård M. A. Ivarsson Y. Tars K. Mannervik B. (2006). Alternative Mutations of a Positively Selected Residue Elicit Gain or Loss of Functionalities in Enzyme Evolution. Proc. Natl. Acad. Sci. United States America 103 (13), 4876–4881. 10.1073/pnas.0600849103 PMC1458763 16549767

[B57] Pettersen E. F. Goddard T. D. Huang C. C. Couch G. S. Greenblatt D. M. Meng E. C. (2004). UCSF Chimera-Aa Visualization System for Exploratory Research and Analysis. J. Comput. Chem. 25 (13), 1605–1612. 10.1002/jcc.20084 15264254

[B58] Pettersson P. L. Johansson A. S. Mannervik B. (2002). Transmutation of Human Glutathione Transferase A2-2 with Peroxidase Activity into an Efficient Steroid Isomerase. J. Biol. Chem. 277 (33), 30019–30022. 10.1074/jbc.M204485200 12023294

[B59] Pettersson P. L. Mannervik B. (2001). The Role of Glutathione in the Isomerization of Δ^5^-androstene-3,17-dione Catalyzed by Human Glutathione Transferase A1-1. J. Biol. Chem. 276 (15), 11698–11704. 10.1074/jbc.M009146200 11152686

[B60] Pollack R. M. (2004). Enzymatic Mechanisms for Catalysis of Enolization: Ketosteroid Isomerase. Bioorg. Chem. 32 (5), 341–353. 10.1016/j.bioorg.2004.06.005 15381400

[B61] Rabahi F. Brûlé S. Sirois J. Beckers J. F. Silversides D. W. Lussier J. G. (1999). High Expression of Bovine Alpha Glutathione S-Transferase (GSTA1, GSTA2) Subunits Is Mainly Associated with Steroidogenically Active Cells and Regulated by Gonadotropins in Bovine Ovarian Follicles. Endocrinology 140 (8), 3507–3517. 10.1210/endo.140.8.6886 10433206

[B62] Raffalli-Mathieu F. Orre C. Stridsberg M. Hansson Edalat M. Mannervik B. (2008). Targeting Human Glutathione Transferase A3-3 Attenuates Progesterone Production in Human Steroidogenic Cells. Biochem. J. 414 (1), 103–109. 10.1042/BJ20080397 18426392

[B63] Raffalli-Mathieu F. Persson D. Mannervik B. (2007). Differences between Bovine and Human Steroid Double-Bond Isomerase Activities of Alpha-Class Glutathione Transferases Selectively Expressed in Steroidogenic Tissues. Biochim. Biophys. Acta 1770 (1), 130–136. 10.1016/j.bbagen.2006.06.015 16934407

[B64] Ran F. A. Hsu P. D. Wright J. Agarwala V. Scott D. A. Zhang F. (2013). Genome Engineering Using the CRISPR-Cas9 System. Nat. Protoc. 8 (11), 2281–2308. 10.1038/nprot.2013.143 24157548 PMC3969860

[B65] Seeley S. K. Poposki J. A. Maksimchuk J. Tebbe J. Gaudreau J. Mannervik B. (2006). Metabolism of Oxidized Linoleic Acid by Glutathione Transferases: Peroxidase Activity toward 13-hydroperoxyoctadecadienoic Acid. Biochim. Biophys. Acta 1760 (7), 1064–1070. 10.1016/j.bbagen.2006.02.020 16624487

[B66] Sinning I. Kleywegt G. J. Cowan S. W. Reinemer P. Dirr H. W. Huber R. (1993). Structure Determination and Refinement of Human Alpha Class Glutathione Transferase A1-1, and a Comparison with the Mu and Pi Class Enzymes. J. Mol. Biol. 232 (1), 192–212. 10.1006/jmbi.1993.1376 8331657

[B67] Škerlová J. Lindström H. Gonis E. Sjödin B. Neiers F. Stenmark P. (2020). Structure and Steroid Isomerase Activity of Drosophila Glutathione Transferase E14 Essential for Ecdysteroid Biosynthesis. FEBS Lett. 594 (7), 1187–1195. 10.1002/1873-3468.13718 31845319

[B68] Stockman P. K. Beckett G. J. Hayes J. D. (1985). Identification of a Basic Hybrid Glutathione S-Transferase from Human Liver. Glutathione S-Transferase delta Is Composed of Two Distinct Subunits (B1 and B2). Biochem. J. 227 (2), 457–465. 10.1042/bj2270457 4004774 PMC1144864

[B69] Suzuki T. Johnston P. N. Board P. G. (1993). Structure and Organization of the Human Alpha Class Glutathione S-Transferase Genes and Related Pseudogenes. Genomics 18 (3), 680–686. 10.1016/s0888-7543(05)80373-8 8307579

[B70] Talalay P. (1965). Enzymatic Mechanisms in Steroid Biochemistry. Annu. Rev. Biochem. 34, 347–380. 10.1146/annurev.bi.34.070165.002023 14321174

[B71] Talalay P. Wang V. S. (1955). Enzymic Isomerization of delta5-3-ketosteroids. Biochim. Biophys. Acta 18 (2), 300–301. 10.1016/0006-3002(55)90079-2 13276386

[B72] Tars K. Olin B. Mannervik B. (2010). Structural Basis for Featuring of Steroid Isomerase Activity in Alpha Class Glutathione Transferases. J. Mol. Biol. 397 (1), 332–340. 10.1016/j.jmb.2010.01.023 20083122

[B73] Thomas J. L. Boswell E. L. Scaccia L. A. Pletnev V. Umland T. C. (2005). Identification of Key Amino Acids Responsible for the Substantially Higher Affinities of Human Type 1 3β-Hydroxysteroid Dehydrogenase/isomerase (3β-HSD1) for Substrates, Coenzymes, and Inhibitors Relative to Human 3β-HSD2. J. Biol. Chem. 280 (22), 21321–21328. 10.1074/jbc.M501269200 15797861 PMC1282470

[B74] Warshel A. (1998). Electrostatic Origin of the Catalytic Power of Enzymes and the Role of Preorganized Active Sites. J. Biol. Chem. 273 (42), 27035–27038. 10.1074/jbc.273.42.27035 9765214

[B75] Wolfenden R. Snider M. J. (2001). The Depth of Chemical Time and the Power of Enzymes as Catalysts. Acc. Chem. Res. 34 (12), 938–945. 10.1021/ar000058i 11747411

[B76] Yabukarski F. Biel J. T. Pinney M. M. Doukov T. Powers A. S. Fraser J. S. (2020). Assessment of Enzyme Active Site Positioning and Tests of Catalytic Mechanisms through X-ray-derived Conformational Ensembles. Proc. Natl. Acad. Sci. United States America 117 (52), 33204–33215. 10.1073/pnas.2011350117 PMC7776779 33376217

[B77] Zhang W. Dourado D. F. Fernandes P. A. Ramos M. J. Mannervik B. (2012). Multidimensional Epistasis and Fitness Landscapes in Enzyme Evolution. Biochem. J. 445 (1), 39–46. 10.1042/BJ20120136 22533640

[B78] Zhang Y. Kolm R. H. Mannervik B. Talalay P. (1995). Reversible Conjugation of Isothiocyanates with Glutathione Catalyzed by Human Glutathione Transferases. Biochem. biophysical Res. Commun. 206 (2), 748–755. 10.1006/bbrc.1995.1106 7826396

